# Interactions of silica nanoparticles with lung epithelial cells and the association to flotillins

**DOI:** 10.1007/s00204-012-0876-5

**Published:** 2012-06-06

**Authors:** Jennifer Kasper, Maria I. Hermanns, Christoph Bantz, Olga Koshkina, Thomas Lang, Michael Maskos, Christine Pohl, Ronald E. Unger, C. James Kirkpatrick

**Affiliations:** 1 Institute of Pathology, University Medical Centre, Mainz, Germany; 2 ikfe GmbH, Institut für klinische Forschung und Endwicklung, Mainz, Germany; 3 Institute of Physical Chemistry, Johannes Gutenberg University Mainz, Mainz, Germany; 4 BAM Federal Institute for Materials Research and Testing, Berlin, Germany; 5 Institut für Mikrotechnik, Mainz, Germany

**Keywords:** Silica nanoparticles, Alveolar-capillary barrier, Lung epithelial cells, Endothelial cells, Endocytosis, Flotillin-1, Flotillin-2, Cytotoxicity, Inflammatory response

## Abstract

Amorphous silica nanoparticles (aSNPs) gain increasing popularity for industrial and therapeutic claims. The lung with its surface area of 100–140 m^2^ displays an ideal target for therapeutic approaches, but it represents also a serious area of attack for harmful nanomaterials. The exact nature of the cytotoxic effects of NPs is still unknown. Furthermore, cellular pathways and the destiny of internalized NPs are still poorly understood. Therefore, we examined the cytotoxicity (MTS, LDH) and inflammatory responses (IL-8) for different-sized aSNPs (30, 70, 300 nm) on our lung epithelial cells line NCI H441 and endothelial cell line ISO-HAS-1. Additionally, colocalization studies have been conducted via immunofluorescence staining for flotillin-1- and flotillin-2-bearing endocytic vesicles. Subsequently, the relevance of flotillins concerning the viability of aSNP-exposed epithelial cells has been evaluated using flotillin-1/2 depleted cells (*siRNA*). This study reveals the relevance of the nanoparticle size regarding cytotoxicity (MTS, LDH) and inflammatory responses (IL-8), whereat the smaller the size of the nanoparticle is, the more harmful are the effects. All different aSNP sizes have been incorporated in flotillin-1- and flotillin-2-labelled vesicles in lung epithelial and endothelial cells, which display a marker for late endosomal or lysosomal structures and appear to exhibit a clathrin- or caveolae-independent mode of endocytosis. Flotillin-depleted H441 showed a clearly decreased uptake of aSNPs. Additionally, the viability of aSNP-exposed cells was reduced in these cells. These findings indicate a contribution of flotillins in as yet unknown (clathrin or caveolae-independent) endocytosis mechanisms and (or) endosomal storage.

## Background

Nanotechnology has made tremendous progress in the last decade concerning industrial applications of nanoparticles (NPs) due to their different features compared to the same material in bulk form (Service 2004). Almost any material can be used to produce engineered nanoparticles for industrial purposes (carbon: lubricants, coatings; titanium dioxide: toothpaste, white paints, sunscreens; silver: textile coating with antimicrobial properties; Ellsworth et al. 2000; Institute of Nanotechnology 2011). In particular, amorphous silica nanoparticles (aSNPs) are widely used for industrial applications. aSNPs are processed in paints and coatings, photovoltaic systems, tyre compounds or electrical and thermal insulation material (AEROSIL^®^, EVONIK Industries, http://www.aerosil.com).

They are also added to products to which humans have direct exposure, for example, cosmetics, toothpaste or even powdered food ingredients to avoid caking (Waters et al. 2009; Borm et al. 2006). Furthermore, aSNPs among others are considered to be a promising tool to develop drug and gene carriers purposed for diagnostic and therapeutical approaches (Liu et al. 2011). In general, nanoparticles are increasingly gaining attention in biomedical research for pulmonary delivery of, that is, insulin or calcitonin (Kawashima et al. 1999; Yamamoto et al. 2005; Zhang et al. 2001). Recent research activity involves the development of nanovehicles loaded with, for example, anti-asthmatic drugs (Surti et al. 2008). On the one hand, the lung is an ideal target organ for drug and gene delivery via nanoparticles for both systemic and local applications (asthma, COPD, cystic fibrosis) due to its enormous surface area of ca. 140 m^2^ and the reduced first-pass metabolism, by which the concentration of an applied drug is drastically reduced before reaching the systemic circulation, not to mention the targeted organ (Hoet et al. 2004; Bailey and Berkland 2009). On the other hand, these characteristics make the lung an ideal target site for accidently inhaled hazardous nanoparticles. Hence, attention must be given to possible health hazards of inhalation of NPs destined for industrial or biomedical applications or targets. The attention should not only be focused on cytotoxic effects. Inhaled nanoparticles may cause unwanted inflammatory responses. What is not known are the exact nature of the cytotoxic effects. Furthermore, cellular pathways of internalized NPs and their eventual distribution and localization within the cells are generally not known. It is known that nanoparticle deposition in the peripheral lung appears to be size dependent. Nanosized particulate matter more likely reaches the deep lung compared to micronsized particles (Bailey and Berkland 2009). Furthermore, the deposition efficiencies of different-sized nanoparticles vary for the different regions of the lung (Oberdorster et al. 2005). The size of a nanoparticle is regarded as a pivotal characteristic factor and closely correlates with its surface area. The surface parameter is regarded as a prevailing factor with respect to the reactivity and cytotoxicity of the nanomaterials (Kreyling et al. 2010).

Numerous studies have shed some light on the toxic effects of silica nanoparticles in general (Sun et al. 2011; Nabeshi et al. 2011) or focused on size-dependent toxic effects of aSNPs (Li et al. 2011; Napierska et al. 2009). However, size-dependent inflammatory responses caused by silica nanoparticles are still poorly understood and may display a more sensitive reaction compared to the standard cytotoxicity tests. The goal of this study was to further characterize the toxic effects and uptake of aSNPs. The cytotoxic effects of different-sized aSNPs, which were exposed to the human lung epithelial cell line NCI H441 (H441) and the microvascular endothelial cell line ISO-HAS-1, were examined using the MTS assay, a test for cell viability and the LDH assay, an assay for membrane integrity. In addition, studies were done to investigate the inflammatory responses (IL-8 release) of H441 and ISO-HAS-1 following treatment with different-sized aSNPs. Furthermore, cellular uptake and location of different-sized aSNPs in H441 and ISO-HAS-1 cells was visualized by means of colocalization experiments via immunofluorescent staining of flotillin-1 and flotillin-2, which display a marker for late endosomal or lysosomal structures and appear to exhibit a clathrin- or caveolae-independent mode of endocytosis (Glebov et al. 2006). Finally, we examined the importance of flotillin-1 and flotillin-2 on cytotoxic effects and uptake behaviour of aSNPs in flotillin-containing and flotillin-1- and flotillin-2-depleted H441 generated by siRNA transfection.

## Materials and methods

### Nanoparticle characterization

Sicastar Red are amorphous silica nanoparticles in aqueous dispersion with nominal diameters of 30, 70 and 300 nm. The particles are loaded with a fluorophore, namely Rhodamine B, which is covalently attached to the SiO_2_ matrix. Sicastar Red is commercially available from micromod Partikeltechnologie GmbH (www.micromod.de). The samples were characterized by angular-dependent dynamic light scattering measurements as described previously (Kasper et al. 2011).

In pure aqueous solution, Sicastar Red particles with the nominal diameter of 30 nm showed a hydrodynamic radius R_h_ of 12.6 nm (corresponding diameter: 25.2 nm; see Table 1), indicating that the particles are in a non-aggregated primary particle state. However, in salt-containing buffer solution such as PBS and in serum-free cell culture medium RPMI, these dispersions got destabilized and the particles partly agglomerated: The average hydrodynamic radius in PBS is around 66 and 58 nm in RPMI. Simultaneously, the μ_2_ values, representing the width of the particle size distribution and thus being a measure for the samples polydispersity, also increased. This leads to the assumption that—despite the increased average hydrodynamic radii—there is still some non-agglomerated material left in the dispersion.

**Table 1:** Characterization of fluorescence-labelled, silica-based nanoparticles

	Water		PBS		RPMI	
	R_h_/nm	μ_2_ (90°)	R_h_/nm	μ_2_ (90°)	R_h_/nm	μ_2_ (90°)
Sicastar Red, 30 nm	12.6	0.10	66.2	0.16	58.1	0.17
Sicastar Red, 70 nm	33.2	0.06	34.1	0.03	31.6	0.03
Sicastar Red, 300 nm	157	0.02	151	0.01	151	0.04

Hydrodynamic radii (Rh, with μ_2_ values as an estimate for the polydispersity) in different aqueous solutions (Milli-Q water, phosphate buffered saline (PBS, without Ca^2+^ and Mg^2+^) and serum-free cell culture medium RPMI) were obtained by dynamic light scattering (angular-dependent measurements)

**Fig. 1:**
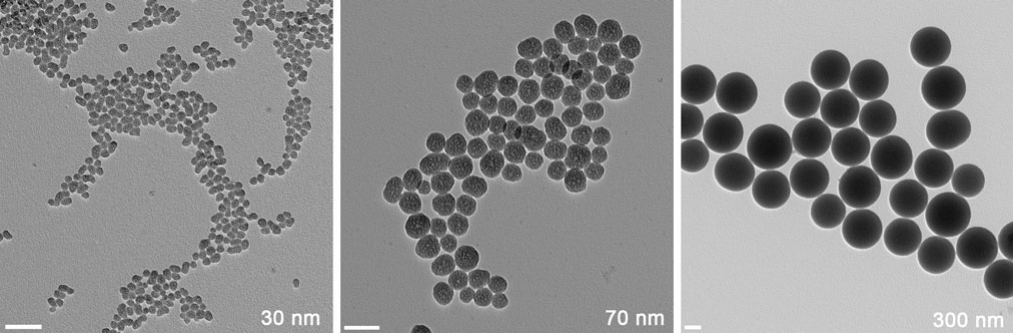
Electron microscopical image of the Sicastar nanoparticles in dry state. The fact that the size of the nanoparticles in the dry state (*D*_*TEM*_) was nearly the same as in solution indicates that the effect of particle shrinking during the preparation of the samples for TEM is minimal. Sicastar 30: *R* = 11.7 ± 1.7 nm (±14.3 %), Sicastar 70: *R* = 32.3 ± 4.1 nm (±12.6 %), Sicastar 300: *R* = 163.7 ± 11.2 nm (±6.9 %). *Scale bar* 100 nm

Contrary to this observation, Sicastar Red with nominal diameters of 70 and 300 nm showed sizes similar to the nominal ones in all aqueous media (i.e. in RPMI: 31.6 nm for the 70 nm aSNP and 151 nm for the 300 nm aSNP). Also, the size distributions of these two particles are very narrow in all cases, even close to true monodispersity, that is, μ_2_ = 0.03 for Sicastar 70 nm and μ_2_ = 0.04 for Sicastar 300 nm, both in RPMI (samples with μ_2_ values < 0.05 are generally considered as monodisperse). Thus, the tendency to form aggregates is limited to the smallest particles in this study. Figure 1 depicts the Sicastar nanoparticles in dry state via TEM (transmission electron microscopy). TEM revealed similar radii of the aSNPs as obtained via DLS (Sicastar 30: *R* = 11.7 ± 1.7 nm (±14.3 %), Sicastar 70: *R* = 32.3 ± 4.1 nm (±12.6 %), Sicastar 300: *R* = 163.7 ± 11.2 nm (±6.9 %). Scale bar = 100 nm).

### Cell culture and nanoparticle exposure

#### Cell culture

ISO-HAS-1 (human microvascular endothelial cell line, (Masuzawa et al. 1999; Unger et al. 2002)) and NCI H441 (human lung adenocarcinoma cell line, purchased from ATCC, ATCC-HTB-174, Promochem, Wesel, Germany) were kept in RPMI 1640 supplemented with 10 % FCS (foetal calf serum), 1 % P/S (Penicillin/Streptomycin). ISO-HAS-1 and H441 were passaged every third day in a dilution of 1:3 until passage 50 and 35, respectively.

#### Monocultures in experimental procedures

Prior to seeding cells, the 96-well plates (TPP, Switzerland) or 8-well μ-slides (ibidi) were coated with 50/300 μl fibronectin for 1 h at 37 °C (5 μg/ml, Roche Diagnostics, Mannheim). The cells were seeded (ISO-HAS-1: 1.6 × 10^4^ cells/well, H441: 3.2 × 10^4^ cells/well) from a confluent culture flask on 96-well plates in RPMI 1640 medium (Gibco) with l-glutamine supplemented with 10 % FCS and Pen/Strep (100 U/100 μg/ml) and cultivated at 37 °C, 5 % CO_2_ for 24 h prior to NP exposure to a confluent cell layer.

#### Nanoparticle application on cell culture

To prevent nanoparticle aggregation, pre-dilutions of the NP dispersions were prepared in pure water (Braun ad injectabilia, Braun Melsungen AG, Melsungen). Due to nanoparticle aggregation in serum-containing medium, serum-free medium was used during 4-h exposure. All dilutions were applied 1:10 in serum-free medium to the cells (96 well and transwells: 10 μl NP dispersion + 90 μl serum-free medium, and ibidi wells: 30 μl NP dispersion + 270 μl serum-free medium). To study cytotoxicity and inflammatory responses, an exposure time of 4 and 4 h/20 h (after 4-h incubation, cells were washed twice with serum-free medium and further cultivated for 20-h period under normal culture condition) was chosen. For colocalization studies, an exposure time of 20 min, 4 and 4 h/20 h was chosen.

#### Cytotoxicity

The viability of the cells was determined using the CellTiter 96^®^ AQueous One Solution Cell Proliferation Assay (MTS, Promega, G3582). After nanoparticle incubation, medium was removed and cells were washed twice with PBS to remove nanoparticle remnants, which can interfere with the MTS reagent. The MTS reagent (MTS stock solution mixed with medium in a ratio of 1:10) was applied on the cell layer for 45 min and transferred into a new plate to measure OD at 492 nm.

#### Membrane integrity

25 μl of the supernatant, collected from nanoparticle exposed H441 and ISO-HAS-1, was used in the LDH CytoTox 96^®^ Non-Radioactive Cytotoxicity Assay (Promega, G1780) to determine lactate dehydrogenase (LDH) release following membrane disruption. To avoid false-positive results in the LDH assay after 4-h exposure, the percentage due to aSNP interferences with the dye has been subtracted from the measurements (i.e. Sicastar Red, 30 nm with 600 μg/ml: 9.8 ± 1.9 % of lysis control).

#### Inflammatory responses

The supernatant of nanoparticle exposed H441 was taken to determine IL-8 release via ELISA (DuoSet R&D, DY208) following the manufacturer′s recommendations. Immunofluorescence (IF) was performed to label endosomal marker proteins flotillin-1 and flotillin-2 (BD, 610821, 610383). After nanoparticle exposure, cells were fixed with methanol/ethanol (endosomal markers) in a ratio of 2:1 for 15 min at room temperature. After fixation, cells were incubated with primary antibody diluted in 1 % PBSA (phosphate buffered saline with 1 % bovine serum albumin) over night at 4 °C. After three washing steps with PBS, cells were incubated with secondary antibody (Alexa Fluor 488) for 1 h at room temperature. Subsequently, cells were washed three times with PBS and nuclei were stained with Hoechst 33342 (Molecular Probes) for 5 min and washed three times. Finally, ibidi μ-slides were mounted with ibidi mounting medium (ibidi, Martinsried).

#### Transfection of H441 with siRNA

siRNAs were purchased from Ambion (Applied Biosystems: Flotillin-1: s19914 (GCAUCAGUGUGGUUAGCUAtt) and Flotillin-2: s5286 (GACUAUAAACAGUACGUGUtt). To prepare the siRNA transfection mixture, siRNA was diluted in serum-free medium and added to the same volume of Gencarrier-1-mix (Gencarrier-1 stock solution diluted 1:25 in serum-free medium) and incubated for 30 min at RT. The final siRNA concentration in the well was 50 nM. The transfection was conducted in parallel to the seeding of cells on tissue culture plastic (TPP). 5 × 10^5^ (6-well plates) or 3 × 10^4^ (96-well plates) cells were seeded in 800 μl (6-well plates) or 40 μl (96-well plates) of low-serum medium (RPMI with 1 % FCS) into a well and siRNA transfection mixture (200 μl for 6-well plates and 10 μl for 96-well plates) has been applied to the cells dropwise and mixed thoroughly. After 7 h of incubation at 37 °C and 5 % CO_2_, 1 ml (50 μl) of high-serum medium (20 % FCS) was added and the cell culture has been continued for further 17 h. After 24 h, the transfection medium was replaced by normal culture medium and the cells were cultured for further 48–72 h to achieve the highest siRNA-inhibition efficiency. Inhibition efficiency of the siRNA was checked via real-time PCR (Power SYBR^®^ Green PCR Master Mix, Applied Biosystems, 436765, Real time PCR machine: 7300 Real Time PCR System, AB). RNA isolation was conducted by means of the RNeasy Mini Kit (Qiagen, 74104) following the manufacturer′s protocol. Reverse transcription of the RNA was performed using the Omniscript Reverse Transcriptase-System (Qiagen, 205113).

### Statistical analysis

From several independent measurements, means and standard deviations were calculated. Analyses are shown as mean ± SD from at least three separate experiments. Testing for significant differences between means was carried out using one-way ANOVA and Dunnett’s multiple comparison test at a probability of error of 5 % (*), 1 % (**) and 0.1 % (***) or two-way ANOVA with Bonferroni′s post-test.

## Results

### Cytotoxicity and inflammatory responses

**Fig. 2:**
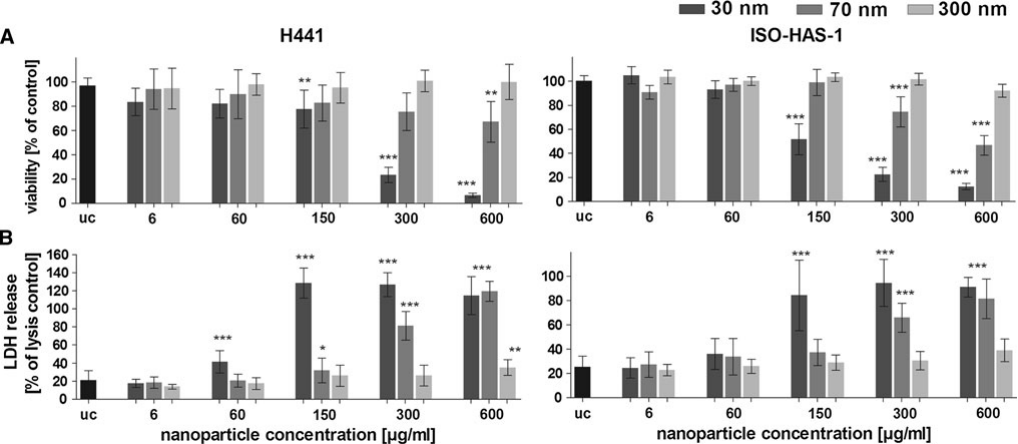
**a** Viability (MTS assay) of H441 following aSNP treatment for 4 h in serum-free medium with different-sized Sicastar Red (30, 70, 300 nm). **b** Lactate dehydrogenase release (LDH) of H441 following aSNP treatment for 4 h with different-sized Sicastar Red (30, 70, 300 nm). Data are depicted as mean ± SD of 3 independent experiments with *n* = 3 samples for each treatment. For statistical analysis, two-way ANOVA with Bonferroni′s post-test was applied. *P < 0.05, **P < 0.01 and ***P < 0.001 compared to the untreated control (uc). 30 nm-sized aSNPs caused a reduced viability and an increased LDH release in H441 compared to the larger-sized aSNPs of 70 and 300 nm

Figure 2a depicts the viability of H441 and ISO-HAS-1 in conventional monoculture after exposure to different-sized aSNPs (Sicastar Red with nominal diameters of 30, 70 and 300 nm, as stated by the supplier) with an incubation period of 4 h at a concentration range of 6, 60, 150, 300, 600 μg/ml (which is consistent with 6.9 × 10^9^; 6.9 × 10^10^; 1.8 × 10^11^; 3.6 × 10^11^; 6.9 × 10^11^ μg/cm^2^) in a volume of 100 μl/well. For both cell lines, significant differences in cytotoxic effects between the three different-sized aSNP were observed (concentration of NPs 150 μg/ml). 30 nm-sized aSNPs caused a reduced viability in H441 (77 ± 13 % of untreated control) compared to the larger-sized aSNPs of 70 and 300 nm, which did not show a significant cytotoxic effect (70 nm: 90 ± 15 %, 300 nm: 96 ± 10 % of untreated control). Higher concentrations (300 and 600 μg/ml) illustrate a clearly increased toxicity of the smaller-sized aSNPs of 30 nm (300 μg/ml: 23 ± 6 %, 600 μg/ml 6.5 ± 2 %) compared to the larger-sized aSNPs of 70 nm (300 μg/ml: 88 ± 18 %, 600 μg/ml: 82 ± 20 %) and 300 nm (300 μg/ml: 102 ± 7 %, 600 μg/ml: 102 ± 11 %). Figure 2b illustrates the LDH release after exposure to different-sized aSNPs (Sicastar Red 30 nm, 70 nm, 300 nm) with an incubation period of 4 h. In H441, LDH leakage occurred for the 30 nm aSNP at a concentration of 60 μg/ml (41 ± 12 % of lysis control) and reached a peak value at an aSNP concentration of 150 μg/ml (128 ± 16 % of lysis control). For 70 nm aSNPs, however, a marginal but still significant LDH leakage was observed at a concentration of 150 μg/ml (32 ± 14 % of lysis control) and the leakage increased with augmented aSNP concentration and reached a total value at 600 μg/ml (119 ± 11 % of lysis control). Incubation with 300 nm aSNPs resulted in a marginal LDH release at a concentration of 600 μg/ml (35 ± 9 % of lysis control). Lower concentrations (300–0.6 μg/ml) did not have an effect on membrane integrity. ISO-HAS-1 showed a similar behaviour concerning MTS and LDH as the H441 after aSNP stimulation, whereat the 30 nm aSNPs caused a decreased viability (52 ± 13 % of untreated control) and an increased membrane integrity (84 ± 29 % of lysis control) at a concentration of 150 μg/ml. However, the larger-sized aSNPs (70 nm) showed a reduced viability (300 μg/ml: 75 ± 12.5 % and 600 μg/ml: 47 ± 8.2 % of untreated control) and an increased LDH release (300 μg/ml; 66 ± 12 % and 600 μg/ml: 82 ± 16 %) at higher concentrations. 300 nm aSNP did not show any significantly altered behaviour compared to the untreated control concerning MTS and LDH even at very high concentration (600 μg/ml).

**Fig. 3:**
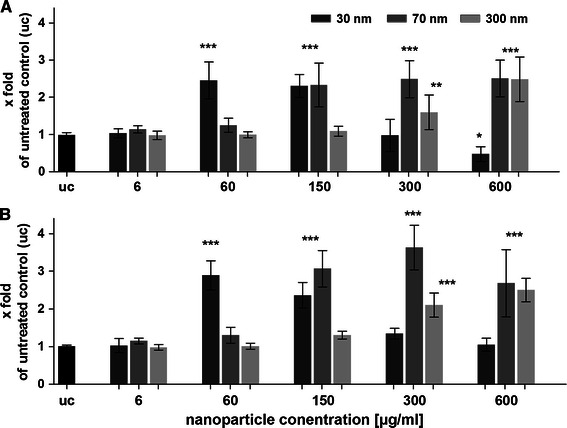
The release of IL-8 was measured after aSNP (Sicastar Red, 30 nm: *dark grey*, 70 nm: *middle grey*, 300 nm *light grey*) exposure of monocultures of H441 (**a**) and ISO-HAS-1 (**b**) on 96-well plates. After 4-h incubation in serum-free medium, aSNPs were removed and the cells were cultivated for further 20 h in serum-containing medium and assayed for IL-8 release. Data are depicted as mean ± SD of 2–3 independent experiments with *n* = 3 samples for each treatment. *P < 0.05, **P < 0.01 and ***P < 0.001 compared to the untreated control. 30 nm-sized aSNPs caused an IL-8 response at lower aSNP concentrations in H441 and ISO-HAS-1 compared to the larger-sized aSNPs of 70 and 300 nm

In Fig. 3, the IL-8 release after 20 h further cultivation of the cells, which were exposed to aSNPs for 4 h, is illustrated. The small-sized aSNPs (30 nm) caused the first peak at a concentration of 60 μg/ml (Fig. 3a, H441: 2.5 ± 0.5 and Fig. 3b, ISO-HAS-1: 2.9 ± 0.39-fold of untreated control, uc) whereas bigger-sized aSNP (70 nm) caused IL-8 release not until a concentration of 150 μg/ml (H441: 2.3 ± 0.58 and ISO-HAS-1: 3 ± 0.48-fold of uc). 300 nm aSNPs resulted in an IL-8 response not until a concentration of 300 μg/ml (H441: 1.6 ± 0.59 and ISO-HAS-1: 2.1 ± 0.32-fold of uc), whereat no effects could be observed for the cytotoxicity assays (MTS, LDH) even with 600 μg/ml. For the 30 nm aSNPs, the IL-8 release is decreasing (i.e. 600 μg for H441: 0.47 ± 0.2 % and ISO-HAS-1: 1 ± 0.17-fold of uc) with increasing aSNP concentration from 60 μg/ml.

### Cellular uptake of different-sized aSNPs

**Fig. 4:**
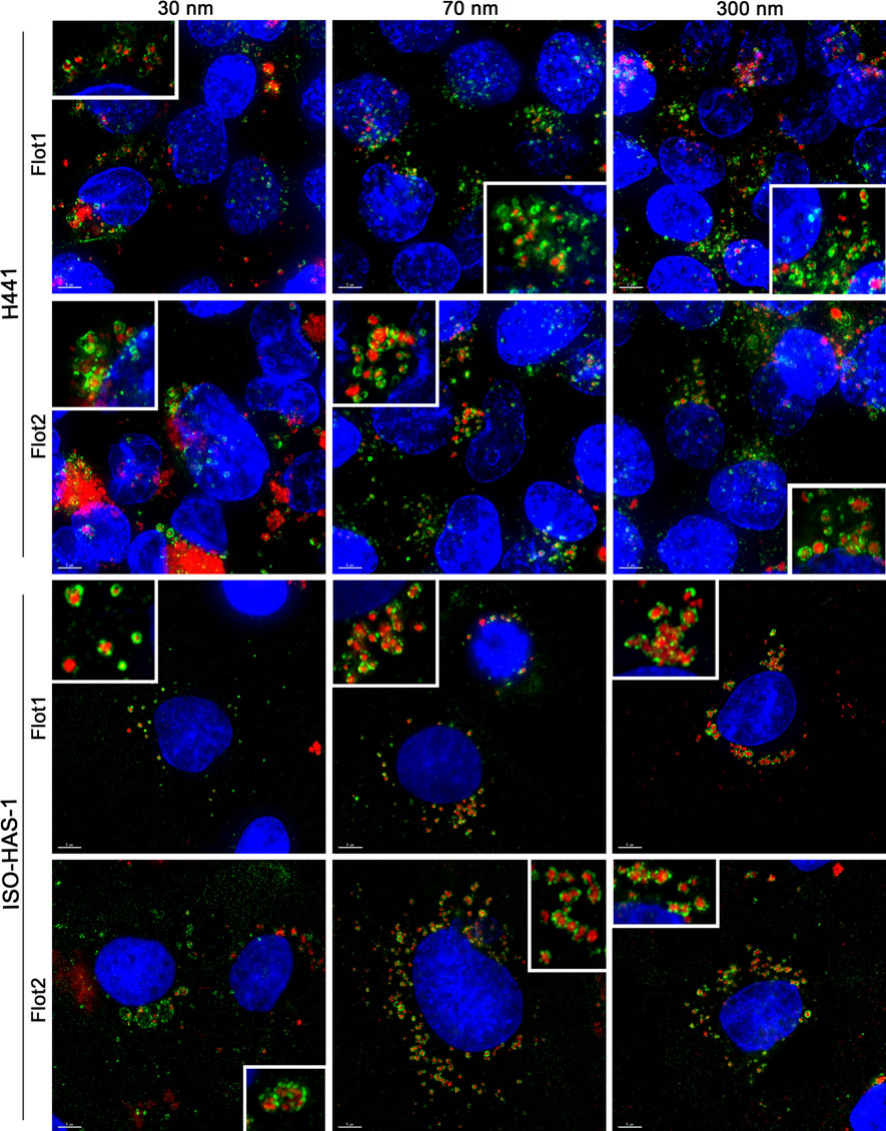
Uptake studies of immunofluorescently (*green signal*: flotillin 1/2) stained H441 and ISO-HAS-1 kept in conventional monoculture and exposed to Sicastar Red at a concentration of 60 μg/ml (30, 70, 300 nm) for 4 h and further 20-h cultivation (*red signal*). A clear incorporation of NPs in flotillin-1- and flotillin-2-containing vesicles (*green signal*) could be detected for all NP sizes. Nuclei are stained using Hoechst 33342 (*blue*). Colocalization of different-sized aSNPs in flotillin-1/2-containing vesicles occurred for all NP sizes. *Scale bar* 5 μm

Figure 4 depicts the uptake and colocalization with flotillin-1/2-containing vesicles of different-sized aSNPs (30, 70, 300 nm). aSNPs of all sizes are incorporated in flotillin-1/2 vesicles.

### aSNP exposure on flotillin-1- and flotillin-2-depleted cells

Transfection efficiency was determined via real-time PCR. After 4 days, the flotillin-1 and flotillin-2 RNA amount was determined. Real-time PCR revealed a flotillin-1/2 RNA reduction to 32 ± 9 % *P* < 0.01 for flotillin-1 and 31 ± 6 % (*P* < 0.01) for flotillin-2 (compared to untransfected control), whereas the negative control (neg) remained unaffected (flotillin-1: 126 ± 17 % and flotillin-2: 93 ± 15 %, *P* > 0.05). In addition, the fluorescence signal of immunofluorescence-labelled flotillin-1/2 decreased in flotillin-1/2-depleted cells compared to the untransfected cells (see fluorescence signals in Fig. 7). Subsequently and prior to experiments with NPs, cytotoxicity of the transfection procedure was determined via MTS. The viability of cells was not significantly affected after negative (92 ± 16 % of untransfected control uc) and flotillin-1/2 (90 ± 18 % of untransfected control) siRNA treatment. Four days after the transfection of H441 cells on 96-well plates with *siRNA*, cells reached a confluent growth and aSNPs (Sicastar Red) were applied to the cells for 4 h in serum-free medium and 4 h followed by 20-h recovery in fresh FCS-containing medium. Concentrations of aSNP ranged from 6 to 300 μg/ml.

**Fig. 5:**
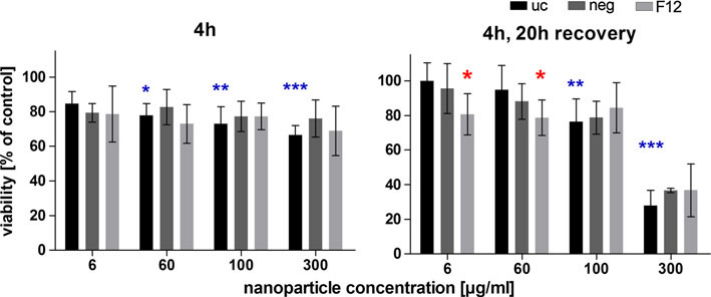
Cytotoxicity (MTS) of flotillin-1- and flotillin-2-depleted H441 following aSNP treatment with 6–300 μg/ml Sicastar Red (30 nm). *uc* untransfected control (no siRNA treatment), *neg* Silencer^®^ Negative Control #1 siRNA and *F12* Silencer^®^Select siRNA for flotillin-1 and flotillin-2 in combination (siRNA concentration: 50 nM). Data are depicted as mean ± SD of 3 independent experiments with *n* = 3 samples for each treatment. For statistical analysis, two-way ANOVA with Bonferroni′s post-test was applied. *P < 0.05, **P < 0.01 and ***P < 0.001 compared to the untreated control of the respective siRNA pre-treatment. *Blue asterisks*: “one-way ANOVA: within the untransfected control”, *red asterisks*: “two-way ANOVA: within a concentration of aSNP”. After 4-h exposure and 20-h recovery, subtoxic concentrations of 6 and 60 μg/ml Sicastar Red showed a significantly reduced viability of flotillin-1/2-depleted cells compared to untransfected cells

**Fig. 6:**
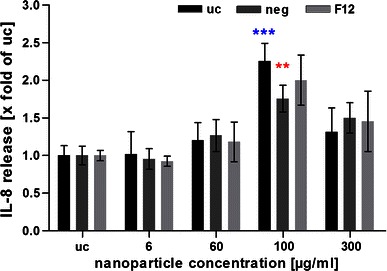
IL-8 release of flotillin-1- and flotillin-2-depleted H441 following aSNP treatment with 6-300 μg/ml Sicastar Red (30 nm in size). *uc* untransfected control (no siRNA treatment), *neg* Silencer^®^ Negative Control #1 siRNA and *F12* Silencer^®^Select siRNA for flotillin-1 and flotillin-2 in combination (siRNA concentration: 50 nM). Data are depicted as mean ± SD of 2 independent experiments with *n* = 3 samples for each treatment. For statistical analysis, two-way ANOVA with Bonferroni′s post-test was applied. *P < 0.05, **P < 0.01 and ***P < 0.001 compared to the untreated control of the respective siRNA pre-treatment. *Blue asterisks*: “one-way ANOVA: within the untransfected control”, *red asterisks*: “two-way ANOVA: within a concentration of aSNP”

**Fig. 7:**
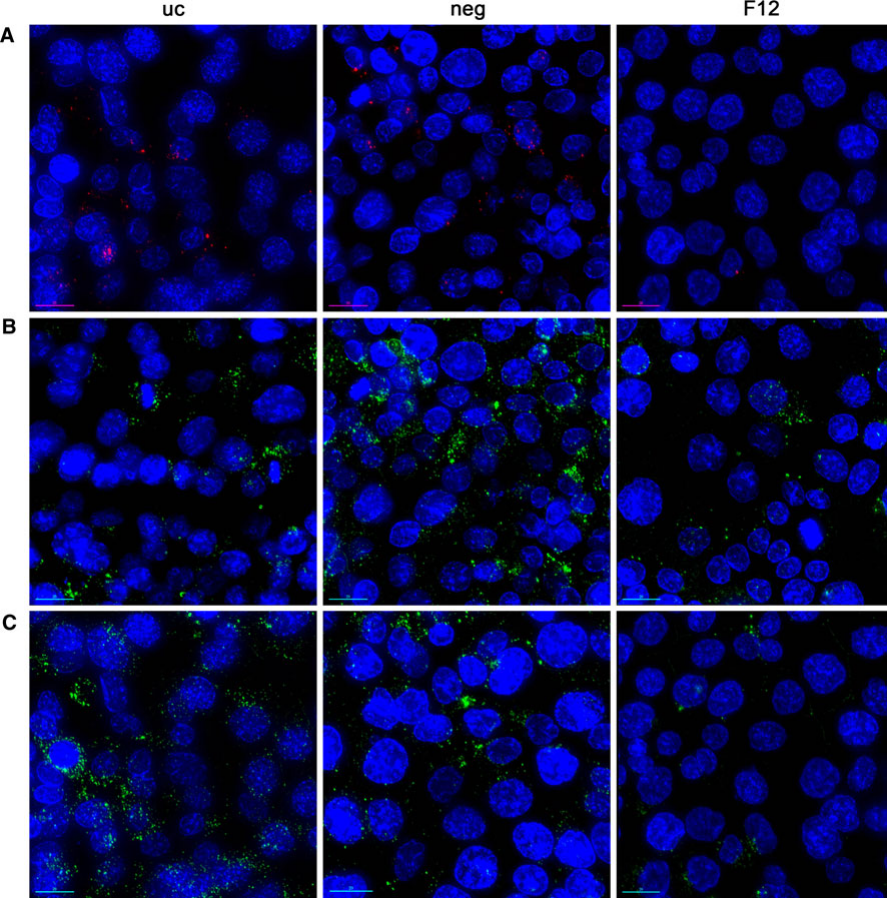
**a** Fluorescence signal of Sicastar Red (30 nm in size, 6 μg/ml) exposed to H441 (*uc* untransfected, *neg* non-targeted siRNA, *F12* siRNA against flotillin-1/2) in conventional monoculture for 4 h in serum-free medium with further 20-h cultivation in fresh serum-containing medium. Furthermore, transfected H441 were stained via immunofluorescence for flotillin-1 (**b**) and flotillin-2 (**c**). Pictures were taken by means of a fluorescence microscope (DeltaVision, Applied Precision). For appropriate comparison, exposure time and intensity scale was equally adjusted. Flotillin-1/2-depleted cells take-up less Sicastar Red than non-targeted siRNA and untransfected cells. Nuclei are stained using Hoechst 33342 (*blue*), *scale bar* 5 μm

**Fig. 8:**
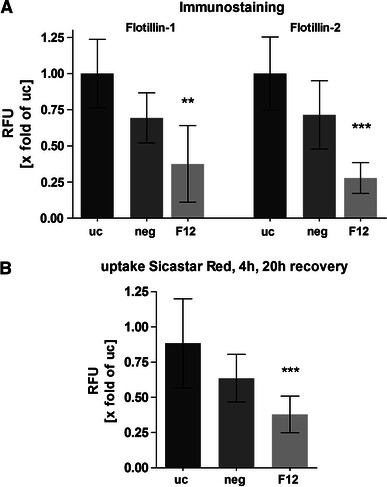
Determination of RFU (relative fluorescent unit related to untransfected control cells) **a** Immunofluorescent staining (IF) for flotillin-1/2 and subsequent RFU measurement. Data are depicted as mean ± SD of 2 independent experiments with *n* = 5 images. **b** uptake quantification of aSNP Sicastar Red (6 μg/ml) in H441 (*uc* untransfected, *neg* treated with non-targeted siRNA, *F12* Flotillin-1/2-depleted cells). Exposure time: 4 h with further cultivation for 20 h in fresh medium. For statistical analysis, one-way ANOVA with Dunnett′s multiple comparison test was applied with *P < 0.05, **P < 0.01 and ***P < 0.00. Flotillin-1/2-depleted cells showed a significant reduction of flotillin-1 and flotillin-2 fluorescence signal and a decreased internalization of Sicastar Red compared to non-targeted siRNA and untransfected cells

After 4 h of aSNP exposure, untransfected H441 showed a significantly decreased viability (78 ± 6.8 % of untreated control) following Sicastar Red (30 nm) exposure at a concentration of 60 μg/ml (see Fig. 5). Viability further decreased with increasing concentration, and at a concentration of 300 μg/ml, a viability of 66 ± 5.5 % was observed, compared to the untreated control. After a 20-h incubation period with fresh FCS-containing medium, the H441 exposed to 60 μg/ml aSNP “recovered” and no significant toxic effect (95 ± 14 %) was detected. Concentrations of 100 μg/ml aSNP displayed a similar effect compared to 4-h exposure (76 ± 13 vs. 73 ± 9.9 %), whereas 300 μg/ml aSNP elicited a further decline in viability (28 ± 8.8 %). Comparing the viability of untransfected and transfected cells after Sicastar Red exposure, differences were detected in a concentration range of 6–300 μg/ml. The non-targeted *siRNA* (neg) did not show any alterations compared to the untransfected cells concerning cytotoxicity after aSNP exposure. Moreover, after an incubation time of 4 h, no significant differences between untransfected and flotillin-1/2-depleted (F12) aSNP-exposed cells were found. Nevertheless, after the 20-h recovery period, low, subtoxic concentrations of aSNPs, 6 and 60 μg/ml, showed significant variances displaying a reduced viability of flotillin-1/2-depleted cells compared to untransfected cells (see red asterisks in Fig. 5). Figure 6 displays the IL-8 release of flotillin-1/-2-depleted and aSNP-exposed H441 for 4 h with further 20-h cultivation in fresh medium. An aSNP concentration of 100 μg/ml resulted in a significant IL-8 release for the untransfected control group (2.55 ± 0.24-fold of untreated control with *P* < 0.0001 within the untransfected control group using one-way ANOVA). For the *siRNA-*transfected cells, an IL-8 peak could also be detected at a concentration of 100 μg/ml (neg: 1.75 ± 0.18 and F12: 2 ± 0.33-fold of untreated control), but for neg significantly lower compared to the peak for the untransfected control group (*P* < 0.01) and for F12 with an increased standard deviation (0.33). Surprisingly, flotillin-1/2-depleted cells took up less aSNP (Sicastar Red) than untransfected cells according to visual assessment of images taken by a fluorescence microscope (Fig. 7). In order to obtain more specific evidence of these findings, the uptake of Sicastar Red was quantified. Since the concentrations of 6 and 60 μg/ml were too low to determine the amount of aSNPs taken up by H441 by spectrophotometric analysis, the quantification of internalized aSNP by H441 was conducted by means of intensity measurements of images made by a wide-field fluorescence microscope (personalDV, Applied Precision, Issaquah, USA). In Fig. 8 A, the relative fluorescent unit (RFU: related to the untreated control = 1) was determined for the fluorescence signal of Sicastar Red, which was incubated at a concentration of 6 μg/ml with H441 in conventional monoculture. Non-targeted siRNA showed a minor, non-significant reduction of fluorescence intensity (neg: 0.64 ± 0.17 %) of incorporated Sicastar incubated for 4 h followed by 20-h recovery in fresh medium. Flotillin-1/2-depleted cells, however, showed a significant decrease of internalized aSNPs according to RFU measurements (flotillin 1/2: 0.38 ± 0.13 %) (see Fig. 8b). Furthermore, quantification studies have been conducted via immunofluorescent staining for flotillin-1/2. RFU measurements of immunofluorescent staining against flotillin-1/2 revealed a significant reduction of flotillin-1 (0.38 ± 0.27 %) and flotillin-2 (0.28 ± 0.1 %) fluorescence signal in flotillin-1/2-depleted H441 cells (Fig. 8a). Non-targeted siRNA transfection resulted in a minor but non-significant reduction of fluorescence intensity of flotillin-1 (0.7 ± 0.17 %) and flotillin-2 (0.71 ± 0.24 %). In summary, flotillin-1/2 depletion results in a reduction of flotillin-1/2 and a decreased uptake of aSNP. Furthermore, although a decreased uptake of aSNPs in flotillin-1/2-depleted cells was detected, an increased toxicity of aSNPs in flotillin-1/2-depleted cells was found.

## Discussion

Using MTT and LDH assays, Napierska et al. (2009) previously demonstrated a particle size-dependent cytotoxic effect in a (supposed) human endothelial cell line (EAHY926) caused by aSNPs. The results of the present study corroborate their findings, as in this study the smaller-sized aSNPs (30 nm) were found to cause greater damage to lung epithelial cells (H441) as determined by the MTT and LDH assays compared to larger-sized (70, 300 nm) aSNPs. Despite the fact that the 30 nm aSNPs showed a slight tendency of aggregation, it still displayed a higher toxicity than larger aSNPs, indicating enough non-aggregated material, which was also corroborated by the high polydispersity index (μ^2^ (90°) = 0.17). In summary, cytotoxicity appears to increase with decreasing particle size. As in Napierska et al. (2009), the dose was expressed as a mass concentration. Choosing the right dosimetry is a double-edged sword. Comparing mass concentrations of different-sized NPs leads to a comparison of different particle numbers. However, a mass concentration of, for example, 60 μg/ml (lowest concentration, for which a cytotoxic effect occurred with 30 nm aSNP) of the 30 nm aSNP matches with a particle number of 2 × 10^12^ particles/ml according to the manufacturer’s specifications, whereas 60 μg/ml of 300 nm aSNP corresponds to 2 × 10^9^ particles/ml (i.e. 1,000× less particles compared to 30 nm aSNP). However, adjusting the 300 nm aSNP to 2 × 10^12^ particles/ml would lead to a mass concentration of 60 mg/ml, which is far beyond any physiologically relevant amount. Additionally, the highest mass concentration of 300 nm that can be applied is 2 × 10^11^ particles/ml. However, this particle number corresponds to a mass concentration of 6 μg/ml for the 30 nm aSNPs, which is beyond the toxic range of the 30 nm aSNP. Moreover, an applied dose of 600 μg/ml and also 60 μg/ml does not simulate realistic exposure conditions as it might occur for, for example, a ceramic worker. These concentrations may only occur for accidently inhaled aSNP and were chosen to display the differences for the different-sized particles. Lison et al. (2008) considered mass concentration, particle number, surface area and also the applied volume of NP dispersion as crucial factors for reliable conclusions on nanoparticle cytotoxicity. In general, since the smaller the nanoparticle the bigger the surface area per mass, the surface area is supposed to be a pivotal factor for the displayed biological activity, as reviewed by Oberdörster et al. (2005).

Inflammatory cytokines such as IL-8 play a pivotal role in the recruitment and regulation of neutrophils and it has been shown by other groups to be involved in the NP-induced inflammatory reaction (Cho et al. 2007; Hetland et al. 2001). Hefland et al. (2001) also dedicated the smallest silica NPs as the most potent and correlated the inflammatory response to the surface area, since the differences between different-sized NPs disappeared when related to equal surface areas. This can be corroborated by the results of this study, whereat the IL-8 release of aSNP-exposed cells (H441 and ISO-HAS-1) is increasing with decreasing particle size (when equal mass concentrations are compared). Furthermore, this study accentuated analysing inflammatory responses as the more sensitive method to examine the harmfulness of nanoparticles compared to cytotoxic assays (MTS and LDH), since significant effects could be observed at aSNP concentrations, where MTS and LDH have not yet indicated any toxic causes.

Furthermore, uptake pathways such as clathrin- and caveolae-mediated endocytosis mechanisms have been investigated via immunofluorescent staining of, for example, clathrin heavy chain, EEA1 (Early Endosome Antigen 1) or caveolin 1. However, no definite uptake of aSNPs via such conventional pathways could be detected (data not shown). However, all different-sized aSNPs were observed incorporated in flotillin-1- and flotillin-2-containing vesicles. Flotillin-1 and flotillin-2 are lipid raft proteins (also called reggie-2 and reggie-1, respectively) and are discussed to be involved in clathrin- and caveolae-independent uptake pathways (Glebov et al. 2006; Ait-Slimane et al. 2009; Langhorst et al. 2008) or represent a marker for late/lysosomal compartments (Dermine et al. 2001; Glebov et al. 2006). Own observations also showed a partly colocalization of both flotillins with LAMP1 or cathepsin D in H441 and ISO-HAS-1 (data not shown).

Since the involvement of flotillins in endocytosis mechanism can be observed in a variety of cell types such as macrophages (J774) (Dermine et al. 2001), epithelial cells (H441 and HeLa (Glebov et al. 2006)) or endothelial cells (ISO-HAS-1) and an incorporation of nanoparticles of different sizes (aSNP: 30, 70, 300 nm) or material such as amorphous silica, organosiloxane particles or poly(ethyleneimine) (further manuscript in preparation), it appears to be a general phenomenon.

Although recent studies emphasize the size of NPs as a critical factor for entering the respective uptake pathways (Vivero-Escoto et al. 2010), in this study the size of aSNPs, ranging from 30 to 300 nm, was not crucial for targeting flotillin-1/2-bearing vesicles in H441 and ISO-HAS-1 (see Fig. 4 for Sicastar Red after 24 h). This supports the findings of Shapero and co-workers (Shapero et al. 2011), who detected an aSNP accumulation (50 and 100 nm in size) in lysosomes of A549 cells after 24 h. The latter group also found a colocalization of aSNPs with early endosomes, multilamellar bodies and multivesicular bodies of A549 cells using electron microscopy, which is a more sensitive method (due to the higher resolution) to detect nanosized material than fluorescence-based detection methods, including immunofluorescence. Besides the size dependency, other factors may also play a crucial role concerning cellular uptake behaviour. On the one hand, a defined surface coating of the nanoparticle is decisive, which is already indicated by several studies focussing on an enhanced uptake and retention of drugs meant for, for example, systemic or asthma treatment using surface-modified nanovehicles (Yamamoto et al. 2005; Surti et al. 2008). On the other hand, nanoparticles, which are dispersed in protein-containing cell culture media, may adsorb proteins at the surface and form a protein corona, which is recognized by the cell despite of the NP itself (Walkey and Chan 2011).

A depletion/reduction of flotillin-1 and flotillin-2 expression in flotillin-1/2 siRNA-treated H441 cells was confirmed by real-time PCR studies. Furthermore, the fluorescence intensity of the immunofluorescent staining of flotillin-1/2 revealed a significant reduction of flotillin-1/2-containing vesicles in flotillin-1/2-depleted H441 cells. In this study, quantification of flotillin-1/2-depleted H441 cells showed a reduced uptake of Sicastar Red compared to non-transfected control cells. This finding may indicate an involvement of flotillin-1/2 in the uptake mechanisms of Sicastar Red. The control cells, which were transfected with Silencer^®^ Negative *siRNA* (neg), also displayed a slight, but not significant reduction of the fluorescence intensity of the aSNP fluorescence signal. Real-time PCR results, however, did not indicate any reduction of flotillin-1/2 expression in non-targeted siRNA-transfected H441. Due to the fact that the transfection procedure using Gencarrier-1, which is a lipoplex-based transfection reagent, may influence endocytosis metabolism, since the lipoplexes follow the clathrin-dependent endocytosis route, non-specific alterations in uptake of aSNPs may be explainable on the basis of these pre-treatments. For this reason, it is essential to carry out appropriate control experiments to verify non-specific influences of pre-treatments, such as transfection reagents.

The mechanisms by which aSNPs exert their cytotoxic effect are still largely unknown. However, there are many possibilities, including damage to the plasma membrane before particles penetrate the cells, intracellular interference after uptake in late or lysosomal structures, and lysosomal escape. Therefore, further experiments were conducted to obtain more insight into these possible pathways. Cytotoxicity of flotillin-1/2-depleted H441 following aSNP (Sicastar Red) exposure was evaluated and compared to non-depleted cells. The toxic influence of aSNPs (Sicastar Red) at low concentrations (6–60 μg/ml) was increased in flotillin-1/2-depleted cells. After 4 h, a similar toxicity was observed for non-transfected, aSNP-treated cells and flotillin-1/2-depleted, aSNP-treated cells. However, after 20 h of recovery, differences between treatments became evident. While non-transfected cells appear to recover at low concentrations (6–60 μg/ml) within 20 h after exposure, the toxic effects of aSNPs persisted in flotillin-1/2-depleted cells. For higher aSNP concentrations (300 μg/ml), similar toxicity was observed for all cellular treatments and time-points. Taking the results of Sicastar Red uptake in flotillin-1/2-depleted H441 into account, which indicated a decelerated uptake of aSNP as seen by decreased fluorescence intensity values, an increase in cytotoxicity appears at first sight to be contradictory. Assuming an involvement of flotillins in at least storage/degradation processes, which is corroborated by the localization of NPs in flotillin-1/2-containing vesicles, even lower concentrations of incorporated aSNP might display higher cytotoxicity in flotillin-1/2-depleted cells compared to the higher amount taken up by the control cells (non-transfected). Glebov et al. also studied the flotillin-dependent endocytosis mechanism by applying several inhibition methods (Damke et al. 1995; Glebov et al. 2006). They stressed the need for careful interpretation of results that are obtained from such inhibition experiments, since evidence already exists that by inhibiting a distinct endocytosis pathway, another pathway may compensate instead (Damke et al. 1995). A variety of controls have to be carried out in order to minimize other possible interpretations. This is also shown in this study, where the control group, which where transfected with Silencer^®^ Negative *siRNA*, concomitant with the flotillin-1/-2 depleted cells, resulted in a significantly reduced IL-8 response after aSNP treatment compared to the untransfected, aSNP-exposed control. To approach more realistic the in vivo situation, it would be recommendable to reproduce this study with the multicellular coculture model of the alveolar-capillary barrier consisting of H441 on top and microvascular endothelial cells (ISO-HAS-1), which already has been used to study nanoparticle interactions (Hermanns et al. 2010; Kasper et al. 2011). However, since the H441 in coculture displays a very tight barrier with well-developed tight junctions, it is challenging to yield an efficient siRNA transfection rate, which is also shown by Hermanns et al. (2010).

In summary, this study attenuates the relevance of the nanoparticle size regarding cytotoxicity and inflammatory responses, whereat the smaller the size of the nanoparticle is, the more potent it causes harmful effects. Furthermore, all different aSNPs sizes have been incorporated in flotillin-1- and flotillin-2-labelled vesicles in lung epithelial and endothelial cells, which display a marker for late endosomal or lysosomal structures and appear to exhibit a clathrin- or caveolae-independent mode of endocytosis. Flotillin-depleted epithelial cells showed a clearly decreased uptake of aSNPs. Additionally, the viability of cells after aSNP exposure was reduced in these cells. These findings indicate a contribution of flotillins in as yet unknown (clathrin or caveolae independent) endocytosis mechanisms and (or) endosomal storage.

## Abbreviations

ANOVA: Analysis of variance
aSNP: Amorphous silica nanoparticles
DLS: Dynamic light scattering
EC: Endothelial cells
Flot1: Flotillin-1
Flot2: Flotillin-2
IF: Immunofluorescence
NP: Nanoparticle
PBSA: Phosphate buffered saline with 1 % bovine serum albumin
Pen/Strep: Penicillin/streptomycin
RFU: Relative fluorescent unit
SNPs: Silica nanoparticles
F12: Flotillin-1/-2 depletion
Neg: Negative *siRNA*
